# PrACTiC: A Predictive Algorithm for Chemoradiotherapy-Induced Cytopenia in Glioblastoma Patients

**DOI:** 10.1155/2022/1438190

**Published:** 2022-01-24

**Authors:** Alireza Amouheidari, Zahra Alirezaei, Stefan Rauh, Masoud Hassanpour

**Affiliations:** ^1^Department of Oncology, Isfahan Milad Hospital, Isfahan, Iran; ^2^Paramedical School, Bushehr University of Medical Sciences, Bushehr, Iran; ^3^Department of Medical Oncology, Centre Hospitalier Emile Mayrisch, Esch, Luxembourg; ^4^Research Center for Molecular and Cellular Imaging, Tehran University of Medical Sciences (RCMCI), Tehran, Iran

## Abstract

**Background:**

Chemotherapy-induced cytopenia is the most frequent side effect of chemoradiotherapy in glioblastoma patients which may lead to reduced delivery of treatment. This study aims to develop a predictive model that is able to forecast the cytopenia induced by temozolomide (TMZ) during concomitant chemoradiotherapy.

**Methods:**

Medical records of 128 patients with newly diagnosed glioblastoma were evaluated to extract the baseline complete blood test before and during the six weeks of chemoradiotherapy to create a dataset for the development of ML models. Using the constructed dataset, different ML algorithms were trained and tested.

**Results:**

Our proposed algorithm achieved accuracies of 85.6%, 88.7%, and 89.3% in predicting thrombocytopenia, lymphopenia, and neutropenia, respectively.

**Conclusions:**

The algorithm designed and developed in this study, called PrACTiC, showed promising results in the accurate prediction of thrombocytopenia, neutropenia, and lymphopenia induced by TMZ in glioblastoma patients. PrACTiC can provide valuable insight for physicians and help them to make the necessary treatment modifications and prevent the toxicities.

## 1. Introduction

Glioblastoma is the most common primary brain tumor in adults [1, 2]. The standard treatment for newly diagnosed glioblastoma is actually the maximal safe resection followed by concomitant chemoradiotherapy with temozolomide (TMZ) followed by adjuvant TMZ [3, 4]. Patients receiving TMZ are at risk of hematologic toxicity (thrombocytopenia, lymphopenia, and neutropenia) during therapy. Currently, monitoring with a weekly complete blood count (CBC) during the course of radiotherapy is proposed to identify the hematologic toxicity [5].

The most frequent hematological side effect of TMZ is moderate to severe thrombocytopenia experienced by 10 to 20 percent of glioblastoma patients [6]. Chemoradiotherapy-induced thrombocytopenia may lead to serious and life-threatening consequences, such as intracranial hemorrhage and gastrointestinal bleeding [7]. Overall, hematologic toxicity induced by TMZ may lead to dose reductions, treatment interruptions, or unexpected termination of treatment, which may have a negative impact on the patient's final treatment outcome [3, 6, 8]. If TMZ-induced cytopenia could be predicted during concurrent chemoradiotherapy, there would be an opportunity to selectively apply approaches to prevent the above-mentioned adverse effects [9].

In recent years, there has been an increasing trend in the application of supervised machine learning (ML) in various fields of oncology [10, 11]. Although ML-derived models have not yet entered into routine clinical practice of oncology, the recent advances have shown their potential to improve the standards of early diagnosis and treatment. The existing studies have used ML algorithms in oncology for oncological risk estimation, lesion detection, image assessment, grading and staging, treatment response assessment, and survival prediction [9]. Successful ML models have the ability to help physicians to reduce adverse effects and increase the probability of positive results and guide their decision on applying adaptive radiotherapy/chemoradiotherapy strategies [12, 13].

In predicting chemotherapy or chemoradiotherapy-induced toxicity, several studies have shown the high performance of ML-based models [14]. Different models have been developed to predict toxicities in sarcoma, breast cancer, and metastatic colorectal cancer [12, 13, 15]. In the field of neuro-oncology, the majority of the few published studies evaluated ML algorithms for image analysis and predicting patient outcomes [16]. An ML approach has been developed by Shibahara et al. to estimate myelosuppression induced by nimustine hydrochloride by analyzing patient blood cell counts prior to treatment of brain tumor patients [15].

To the best of our knowledge, no published study has investigated ML-based models to predict the hematologic toxicity during concomitant TMZ and radiotherapy in glioblastoma patients. This study aims to develop an ML-based model to predict treatment-induced thrombocytopenia, granulocytopenia, and lymphopenia in glioblastoma patients receiving radiotherapy plus concurrent TMZ.

## 2. Method

### 2.1. Patients Characteristics and Treatment Protocol

This study was scientifically and ethically approved by Isfahan Milad Hospital Research Committee (project code: IMH-9961) in accordance with Iranian ministry of health regulations on ethics in biomedical research.

We retrospectively reviewed the medical records of 18 to 55-year-old patients with newly diagnosed glioblastoma who were referred to the oncology department between 2018 and 2020.

Then, all of the data was anonymized. The eligible patients were diagnosed with glioblastoma according to pathological criteria, and after surgical resection or stereotactic biopsy, they completed concurrent chemoradiotherapy with TMZ (3D conformal radiotherapy to a total dose of 60 Gy (2-Gy, 30 fractions) plus daily TMZ (75 mg/m^2^/day)). Finally, the data of 128 eligible patients (55 males and 78 females) were considered as the dataset of this study.

### 2.2. Data Organization

A baseline complete blood test (CBC) had been performed before the start of treatment and after that on a regular weekly schedule during the whole chemoradiotherapy course. All CBCs were performed in the hospital laboratory with Veterinary Auto Hematology Analyzer, VH -22 (Labomed Inc., LA, USA). Platelet (PLT), white blood cells (WBC), absolute lymphocytes, and neutrophil counts (ALC and ANC, respectively) plus hemoglobin (HGB) levels were extracted from patients' documents. Hematologic toxicity was graded according to the National Cancer Institute Common Terminology Criteria for Adverse Events version 5.0 (CTCAE v5.0) (ref) as follows: thrombocytopenia grade 1 (150–75/*μ*L), grade 2 (75–50/mm^3^), grade 3 (50000–10000/mm^3^), grade 4 (<25000/mm^3^); neutropenia grade 1 (2000–1500/mm^3^), grade 2 (1500–1000/mm^3^), grade 3 (1000–500/mm^3^), grade 4 (<500/mm^3^); lymphopenia grade 1 (1000–800/mm^3^), grade 2 (800–500/mm^3^), grade 3 (50–200/mm^3^), grade 4 (<200/mm^3^) [17].

### 2.3. Algorithm and Training Dataset

To create an appropriate data set for training and testing our model, we applied the following steps.

The PLT, WBC, ALC, ANC, HGB, and ANC to ALC ratios of all 6 weeks for any patient were extracted from the recorded files. Then, the difference of any of the above parameters between every two consecutive weeks was calculated. Next, the toxicities occurred in the two previous weeks and the above-mentioned difference were considered as the input data to predict the grade of toxicity in the following week. After that, the toxicities were categorized into two classes: class 0 was assigned to grades 0 to 2 and class 1 to grades 3 and 4, respectively. The reason for considering these two classes was that since severe toxicities (grade 3 or 4) are clinically much more critical than others and can affect the treatment cycle; we aimed to predict these severe conditions. In addition, when grades 3-4 toxicity was observed in several consecutive weeks for a patient, only data corresponding to the onset week was entered the training data to prevent obtaining high fake accuracy results. It is worth noting that removing the data related to the mentioned weeks reduces the accuracy, but the obtained accuracy after this removal is much more reliable.

Therefore, in total, 21 input features and one output feature, that is, the class of the future toxicity for either thrombocytopenia, lymphopenia, or neutropenia toxicities, have been imported to train and test our developed ML model.

It is important to mention that gender, age, tumor size, and tumor location were not significantly different between class 0 and class 1 groups.

Then, the above-explained dataset was utilized for training different ML algorithms. Since the data set was not large enough, we used 50-fold cross-validation to avoid bias-induced inaccuracy in predicting the classes. Due to the imbalanced data set, misclassification costs depending on the class proportions were also applied in training the models. In addition to this strategy, we have slightly changed the value of the weighting factors around the class proportion to find the optimum value of the factors that results in the best model performance. Finally, the results of the models with the accuracy and true positive (TP) higher than 70% for both class 0 and class 1 including RUS-Boosted trees, linear discriminant, boosted tree, and naïve Bayes have been obtained. According to these results, in the prediction part of the final version of the PrACTiC algorithm, we have selected RUS-boosted trees model for thrombocytopenia and neutropenia and naïve Bayes model for lymphopenia toxicity prediction. All of the above parts have been implemented in MATLAB 2020b.

## 3. Results

**Figure 1:**
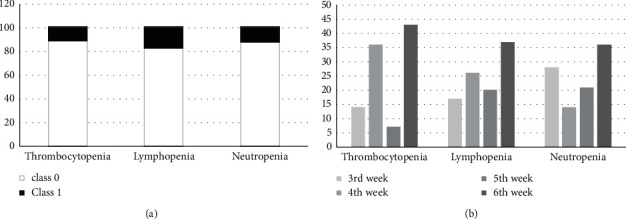
(a) The distribution of class 0 and 1 of cytopenia in all data sets. (b) The percentage of onset week in class 1 cytopenia.

Figure 1(a) shows the frequency of classes 0 and 1 and the onset week of class 1 thrombocytopenia, lymphopenia, and neutropenia.  Figure 1(b) shows the distributions of different grades and the onset of grades 3-4 cytopenia in the final training set.

**Figure 2:**
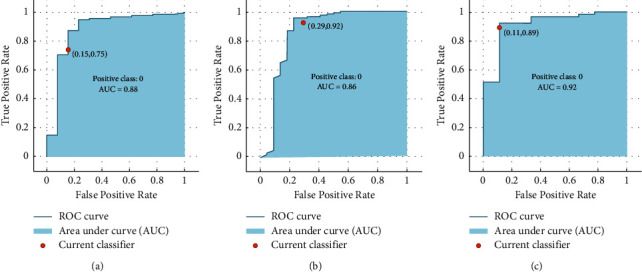
(a) AUC of thrombocytopenia (misclassification cost 10), 1 (b) lymphopenia (misclassification 4), and 1 (c) neutropenia (misclassification cost 11), predicted by RUS-boosted model.

Table 1 shows the true positive (TP), accuracy, and area under the curve (AUC) of the performance of different ML models to predict thrombocytopenia, neutropenia, and lymphopenia. It is worth mentioning that as an incorrect prediction of the occurrence of toxicity may have a high negative impact on the treatment outcome, correct prediction (true positive) of class 1 is much more important than the accuracy and AUC of a model; as a result, we select those models that have shown better performance in predicting class 1. As it is presented in Table 1, only RUS-boosted trees model shows a relatively good performance in predicting thrombocytopenia and neutropenia, while lymphopenia, in addition to RUS-boosted, linear discriminant, boosted tree, and naïve Bayes has shown a good performance (Figure 2(a)–2(c)). The maximum TP was 92%, 78%, and 89% and the maximum accuracy was 85.6%, 88.7%, and 89.3% for the prediction of thrombocytopenia, lymphopenia, and neutropenia, respectively.

## 4. Discussion

### 4.1. TMZ and Hematologic Toxicity

Our results show that the accuracy of PrACTiC achieves 85.6%, 88.7%, and 89.3% with true positives of 92%, 78%, and 89% in predicting thrombocytopenia, lymphopenia, and neutropenia, respectively. These prove that PrACTiC is able to provide the accurate prediction of thrombocytopenia, neutropenia, and lymphopenia toxicities induced by TMZ in glioblastoma patients. Therefore, PrACTiC can provide valuable insight for physicians about the upcoming hematologic toxicities. The insight can be used to make the necessary treatment modifications and prevent the toxicities in glioblastoma patients.

Hematological toxicity of concurrent chemoradiotherapy for glioblastoma patients remains a highly pertinent issue for clinicians. This treatment-induced cytopenia may result in treatment impairment which finally leads to decreased survival and decline in the quality of life of these patients [18–20].

There are several published pieces of research utilizing ML methods for treatment-induced toxicity prediction in oncology [21–25]. To the best of our knowledge, this study is the first study applying ML models to predict hematologic toxicity of concomitant chemoradiotherapy with TMZ in glioblastoma patients [21]. We have designed an ML model that shows relatively good performance to predict the thrombocytopenia, neutropenia, and lymphocytopenia in glioblastoma patients.

The frequency of grades 3-4 cytopenia and the onset of grades 3-4 cytopenia in this study, presented in Figure 1, is in good agreement with the other reports [6]. As explained previously, correctly predicting class 1 is much more important than the correct prediction of class 0. The importance of class 1 correct prediction leads us even to accept some incorrect predictions for this class.

The main finding is that among the trained models, the random undersampling- (RUS-) boosted model showed high predictive results for all types of cytopenia. This can be explained regarding the intrinsic characteristics of this model, which made it practical to be applied for imbalanced data sets. For clarification, RUS part removes examples (randomly) from the majority class until the desired balance is achieved. This algorithm combines random undersampling with boosting, resulting in improved classification performance when training data is imbalanced [26]. Consequently, this technique is the most straightforward method for training an imbalanced dataset. However, linear discriminant, naïve Bayes, and boosted tree also showed high accuracy in predicting lymphopenia. Considering different misclassification costs for these models has enabled them to overcome the imbalanced data set problem. Due to this, they have also shown a relatively good predictive performance in this study.

Several studies have used ML models to predict chemotherapy-induced toxicities in different cancers. In a study on the patients with rhabdomyosarcoma receiving IVA chemotherapy, Cuplov et al. applied machine learning analysis using a gradient boosting regression technique to predict the ifosfamide induced hematological toxicities as a function of neutrophils and platelets initial levels and the initial ifosfamide dose [12]. Oyaga-Iriarte et al. developed an ML model that quite accurately predicted the irinotecan-induced high-grade leukopenia, neutropenia, and diarrhea in metastatic colorectal patients treated with chemotherapy. They utilized backward stepwise logistic regression (BSLR), random forest, and support vector machine (SVM) [13]. In another study, Cho et al. utilized the ML models including SVM, decision tree, XGboosting, and artificial neural network to predict the febrile neutropenia in breast cancer patients undergoing taxane-based chemotherapy [27].

In comparison with the above-mentioned studies, this study has some advantages that are listed in the following:

- PrACTiC algorithm is able to predict the toxicity one week before; so, oncologists may consider some treatment modification or apply different strategies to avoid toxicities which help the patient complete the whole chemoradiotherapy course. Therefore, PrACTiC can be used during TMZ regimen to avoid toxicities. In contrast to ours, Wojcieszynski et al. predicted cytopenia 90 to 180 days after treatment rather than during chemoradiotherapy [28].
- The sample size of this study was much larger (128 patients) than other studies. Other studies used a relatively small dataset, ranging within 20–34, to predict cytopenia during or after chemoradiotherapy or chemotherapy [12, 13, 15]. Therefore, our reported results seem more reliable than other similar studies.
- Most of the existing studies have considered regression-based models to predict treatment-induced toxicity [13, 15, 27]. While regression analysis is an excellent tool in analyzing observations and drawing conclusions, in most cases in which data availability is skewed, generalization and consequently cross-platform application of the derived models may have some limitations [29, 30]. Regression and classification are categorized under the same umbrella of supervised machine learning, but the output variable in the regression is numerical or continuous, while that for classification is categorical or discrete [31]. So, there is an inevitably intrinsic error in fitting data with the model because of making continuous output [32]. Considering these points, we have considered our problem as a classification problem. The most important advantage of this consideration is achieving higher and more reliable accuracies in comparison with regression models.

### 4.2. Clinical Impact

Chemoradiotherapy with TMZ prolongs the overall survival of patients with glioblastoma; accordingly, the development of severe thrombocytopenia during the course of treatment may be accompanied by treatment interruption or early termination that negatively affects survival [33]. Traditionally, the routine practice in case of developing thrombocytopenia is to discontinue the TMZ regimen and wait for the recovery of platelet count to the normal levels. However, some new strategies for prophylaxis and treatment of thrombocytopenia have shown promising results in recent studies. The PLATUM phase II trial showed the efficacy of the thrombopoietin receptor agonist Romiplostim for the prevention and treatment of TMZ-induced thrombocytopenia in glioblastoma patients [34].

Decreased neutrophil and lymphocyte counts during concomitant TMZ and radiotherapy can increase the probability of developing opportunistic infections or even febrile neutropenia. Fortunately, in the current practice, granulocyte colony-stimulating factor (GCSF) and new generation antibiotics are available that can effectively treat and prevent leukopenia, so the efforts to predict neutropenia and lymphopenia during chemoradiation are of great value [35].

It is worth bearing in mind that there are some studies evaluating the adding of bevacizumab to the TMZ and conventional or hypofractionated radiotherapy in patients with newly diagnosed glioblastoma. In such circumstances, considering the added risk of hemorrhage by bevacizumab, the prediction of thrombocytopenia will be of clinical importance [36] that gives this study a considerable clinical value.

If the predicted onset of grades 3-4 toxicity would be in the last week of chemoradiotherapy, it may have no major effect on the completeness of the treatment course but raise the alarm that such patients must be monitored more closely and cautiously in the weeks between the termination of chemoradiotherapy and the beginning of adjuvant chemotherapy to prevent serious complications related to thrombocytopenia or neutropenia [6, 37].

### 4.3. Limitations and Recommendations for Future Studies

This study has some limitations. The first limitation is the imbalanced data set problem which restricted us from utilizing other ML models in predicting hematologic toxicity. The second limitation was the possibility of underestimating the number of grades 3-4 thrombocytopenia. It is widely accepted that automation in hematology is still very controversial in cases of thrombocytopenic patients especially in the presence of interference from nonplatelet particles or platelet abnormalities [38]. Recent studies mainly focused on the counts of low levels of platelets and demonstrated that automated counts were not accurate in severely thrombocytopenic samples [39]. Different values of systematic errors with the maximum value of 25% have been reported for platelet automated counters that should be considered in the platelet counts [38]. The third limitation of the current study was the lack of detailed data of radiotherapy dosimetric parameters, exact information of corticosteroids, and antiepileptic drugs type and doses. Studies have reported some degree of radiation-induced lymphopenia related to the irradiated volume [40, 41]. Also, the effect of the new and old generation of antiepileptic drugs on cytopenia has been shown in glioblastoma patients [8, 42]. So, the dose-volume histogram (DVH) information, the dosage of antiepileptics and corticosteroids can be added as extra features to the PrACTiC algorithm.

For future works, we plan to set a confident interval between grade 2 and grade 3 of thrombocytopenia to help us to be more confident in reporting the class “0” and class “1” in cases that have the platelet counts close to the boundary between grade 2 and grade 3 of thrombocytopenia. For clarification, we are going to consider a probabilistic model instead of the deterministic approach used in this study. Having considered this approach, we can estimate the probability of grade 3 compared to grade 2 thrombocytopenia, depending on how far the platelet count closes to the boundary between these two grades. Adding more patients and treatment-related features to the PrACTiC, and evaluation of this tool in the glioblastoma patients receiving adjuvant TMZ is highly encouraged. Since chemoradiotherapy with TMZ is also used for management of anaplastic astrocytoma, oligodendroglioma, and some of the patients with low-grade glioma [43–45], utilizing PrACTiC for these purposes is also recommended.

Altogether, the model designed and developed in this study (PrACTiC) showed promising results in the accurate prediction of thrombocytopenia, neutropenia, and lymphopenia associated with concurrent radiotherapy and TMZ in newly diagnosed glioblastoma patients. PrACTiC gives the medical practitioners prior knowledge about the grade of toxicity that a patient might suffer in the coming week with high accuracy and, thus, can serve as a great assistant to the clinicians for prophylaxis' monitoring and treatment of hematologic toxicities and to make the necessary treatment modifications, accordingly.

## Figures and Tables

**Table 1:** The true positive of each class and accuracy of thrombocytopenia, neutropenia, and lymphopenia predicted by different machine learning models.

Toxicity type	Misclassification cost	Model	TP-class 1	TP-class 0	Accuracy	AUC
Thrombocytopenia	10	RUS boosted	75	85	**85.6**	0.88
	11		**92**	73	74.8	0.87
	12		85	73	73.9	0.84
Lymphopenia	4	Naïve bayes	**78**	79	78.9	0.83
		RUS boosted	71	84	81.7	0.80
		Naïve bayes	75	79	78.2	0.83
	5	Linear discriminant	71	70	70.4	0.75
		Naïve bayes	75	76	76.1	0.83
	6	Boosted tree	71	92	**88.7**	0.86
		RUS boosted	71	81	79.6	0.81
Neutropenia	9	RUS boosted	**89**	88	88	0.94
	10	RUS boosted	**89**	89	**89.3**	0.92
	11	RUS boosted	**89**	88	88	0.96

Bold values show the highest accuracy of toxicity prediction.

## Data Availability

The data that support the findings of this study are available from the corresponding author upon reasonable request.
